# Inpatient and outpatient treatment for acute malnutrition in infants under 6 months; a qualitative study from Senegal

**DOI:** 10.1186/s12913-019-3903-x

**Published:** 2019-01-25

**Authors:** Tabitha D. van Immerzeel, Maty D. Camara, Indou Deme Ly, Rosemarijn J. de Jong

**Affiliations:** 1 Centre Médico-Social Keru Yakaar, Dakar, Senegal; 2 Head of department Nutrition and Alimentation at the Ministry of Health, University Cheikh Anta Diop, Dakar, Senegal; 3 Albert Royer Children’s Hospital, Dakar, Senegal; 4 KIT, Amsterdam, The Netherlands

**Keywords:** Acute malnutrition <6 months, Young infant feeding, Infant malnutrition, Treatment, Outpatient, Inpatient

## Abstract

**Background:**

Treatment of acute malnutrition in infants under 6 months is a relevant topic regarding the global problem of maternal and child malnutrition. While treatment for older age groups has shifted more towards an outpatient, community based approach, young infants are mostly treated in hospital. This study aims to describe barriers and facilitators for outpatient and inpatient treatment of malnourished infants under 6 months in Senegal.

**Methods:**

This qualitative descriptive study uses in-depth interviews with health workers and focus group discussions with mothers of malnourished infants, conducted from June to September 2015 in two case clinics. In data analysis, Collins’ 3 key factors for a successful nutrition program were used as a theoretical framework: access, quality of care and community engagement.

**Results:**

Within Collins’ 3 key factors, 9 facilitators and barriers have emerged from the data. Key factor access: Outpatient care was perceived as more accessible than inpatient concerning distance and cost, given that there is a milk supplement available. Trust could be more easily generated in an outpatient setting. Key factor quality of care: The cup and spoon re-lactation technique was efficiently used in outpatient setting, but needed close supervision. Basic medical care could be offered to outpatients provided that referral of complicated cases was adequate. Health education was more intensive with inpatients, but could be done with outpatients. Key factor community engagement: The community appeared to play a key role in treating malnourished young infants because of its influence on health seeking behaviour, peer support and breastfeeding practices.

**Conclusions:**

Outpatient care does facilitate access, provided that an affordable milk supplement is available. Quality of care can be guaranteed using an appropriate re-lactation technique and a referral system for complications. The community has the potential to be much engaged, though more attention is required for breastfeeding education. In view of the magnitude of the health problem of young infant malnutrition and its strong relationship with breastfeeding practices, an outpatient community-based treatment approach needs to be considered.

## Background

Improving nutritional status with a focus on maternal and child malnutrition is a perennial global health priority, as formulated in the United Nations (UN) Sustainable Development Goals [1]. Acute malnutrition (AM) or wasting, defined as weight for height z-score <-2, is the most dangerous form of malnutrition and has a prevalence in West and Central Africa of 23.9% in children under the age of 5 [2]; 4% suffer from severe acute malnutrition (SAM) [2], as defined by a weight for height z -score <-3 [3]. Management of SAM has recently shifted from inpatient treatment to a predominantly outpatient, community-based approach in many countries (as in Senegal) for children from 6 months to 5 years [4, 5]. The introduction of the Mid Upper Arm Circumference (MUAC) as a screening tool to detect acute malnutrition in the community [6, 7] and a ready-to-use therapeutic food (RUTF) were important factors contributing to this change [6].

Malnutrition in infants under 6 months of age is assumed to be equally prevalent as in older children, although data are hard to obtain [8, 9]. The treatment approach for this age group has not made the shift to outpatient treatment, because both the screening tool as well as nutritional supplement are unsuitable [10, 11]. The revised World Health Organisation (WHO) treatment guideline for infants <6 months suffering from SAM recommends (diluted) F-100 milk as a supplement using the supplementary suckling (SS) method [5]. Effective breastfeeding is mentioned as a treatment objective. It is noteworthy that the guidelines state that outpatient care is possible in uncomplicated cases, but practical applications are lacking [5].

The MAMI (Management of Acute Malnutrition in Infants under 6 months) Project reports that current inpatient programs struggle in terms of resources (time, space and staff) to give young infants the necessary treatment and counselling. In addition, population coverage of such programs is currently low [8]. Results from the MAMI Project has shown that outpatient treatment for acute malnutrition of infants <6 months is indeed possible [8, 12] and authors call for more research on this approach. In addition, Angood & Mcgrath [13] published a series of 15 detailed research priorities on the management of acute malnutrition in infants <6 months.

In Senegal, the prevalence of acute malnutrition under 5 years of age is 9.1%, with 5.4% among those <6 months [14]. A recent study in Senegal revealed several socio economic determinants of malnutrition [15]. Although the country is not in food crisis, food security is low in certain regions due to short rainy seasons and low agricultural productivity [15]. The educational level of heads of households is low, with 64% of them either not able to read or write or having just had koranic education [15]. Obstetric causes, such as maternal anaemia (64 % of pregnant women) [16] and low birth weight (up to 29%) [17] also contribute to infant malnutrition. There is a reported 25.6% unmet need for family planning services and about one in five pregnancies occur within 2 years of the previous delivery [14]. The rate of exclusive breastfeeding is low (38%) among infants 0-6 months in Senegal [14, 18] and some culturally determined young infant feeding practices might play a role [19], although research is limited. Treatment of AM of infants <6 months is mainly done in an inpatient setting [20].

This study aims to qualitatively describe facilitators and barriers that are of importance in either inpatient or outpatient treatment of infants under 6 months suffering from acute malnutrition in a semi-urban setting in Senegal. Out of Angood’ and Mcgrath’s [13] proposed research questions, numbers 3 and 8 will be elaborated. Question 8 is: “What are the main barriers to existing inpatient interventions for infants <6 months with AM and how might they best be addressed?”. Question 3 is: “What are the priority components (facilitators) of a package of care for outpatient treatment of infants <6 months with AM?”

## Methods

### Study design

This is a descriptive qualitative study with elements of the content analysis design [21]. Collins et al.’s 3 key issues of community-based management of SAM were used as a theoretical framework [22]. Collins and colleagues describe 3 essential factors contributing to the effectiveness of outpatient care in malnourished children. First, care must be accessible and affordable. Secondly, good quality medical care with simple sustainable protocols is necessary. Third, community engagement is needed to help people understand the health problem and accept the services provided [22]. These 3 factors, known for treatment of SAM in children older than 6 months, were used to gain insight about the age group under 6 months. Data were collected using semi-structured individual in-depth interviews (IDI) with health workers as well as focus group discussions (FGD) of mothers visiting nutrition programs, conducted in each clinic from June to September 2015. Both IDIs and FGDs were done for triangulation purposes. Ethical clearance was obtained from the national ethics committee of Senegal (Comité National d’Ethique pour la Recherche en Santé, code: SEN15/30) and at the Royal Tropical Institute in The Netherlands. This report follows QCORECQ reporting guidelines [21].

### Setting

The study took place in two private, non-profit clinics in the capital of Senegal. Both clinics have a long history of treating malnourished children, including infants <6 months with SAM. Each have a large catchment area, serving a mainly urban population. One clinic, called St Martin, has an inpatient approach, and uses diluted F-100 as a milk supplement. The other, Keru Yakaar (House of Hope in the local language), treats infants in an outpatient setting using infant formula offered at a reduced price. Cup and spoon feeding is the refeeding technique in both clinics. Medical treatment, including a broad-spectrum antibiotic, is also administered along with some basic laboratory analyses. Teaching sessions and individual supervision of mothers of malnourished young infants are part of the rehabilitative approach.

### Research team and reflexivity

IDIs and FGDs were conducted by the first author, a female Dutch medical doctor who had worked at Keru Yakaar for 3 years. She interviewed the health workers, some of whom were her colleagues. The IDIs were conducted in French. She was not involved fulltime in the nutrition program, so she did not know all the women participating in the FGD. She knew the basics of the local language (Wolof), sufficiently to follow and moderate the conversation during FGD. To gain the maximum amount of information from the mothers, a Senegalese nurse involved in the nutrition program at Keru Yakaar served as a research assistant and translated all information and conversation into the local language during each FGD. A male Senegalese medical student was present at each IDI and FGD to audio record the data. He did not take part in the conversations.

### Data collection

Similar open-ended interview questions were used for the IDIs and FGDs. They focused on the 3 key aspects of treatment of acute malnutrition among infants under 6 months of age (access, quality of care and community involvement). For example an IDI question about access: “What do you think will make care for mothers with an infant with AM accessible and what could be barriers?” Additional questions were added to further clarify the answer. The interview guide was pilot tested with one health worker and adapted accordingly. IDIs and FGDs were audio recorded using a phone and later transcribed by the medical student.

**Fig. 1:**
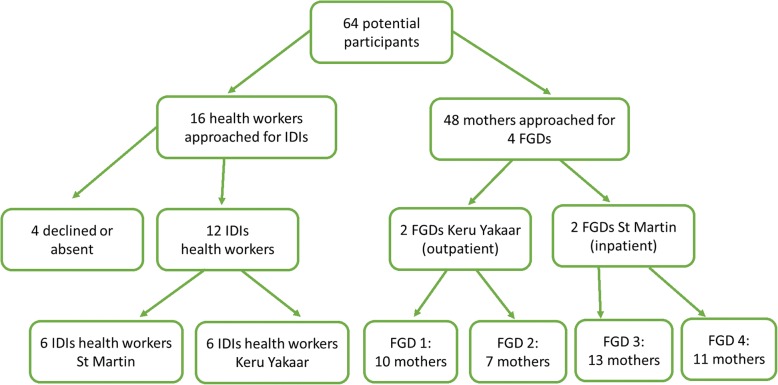
Sampling flow chart IDIs and FGDs

For IDIs, all the health workers on staff in both clinics and involved in the two nutrition programs (nurses, nurse assistants, midwives, and doctors) were approached and invited to participate in the study (convenience sampling); 4 declined (Fig. 1). All participating health workers signed a consent form before the IDI was conducted. Participants were interviewed in French, at their work place, at a time agreed upon by the interviewer and the participant. Each IDI took about 20-30 minutes. Data saturation was recognized with 12 interviews. Health workers were not identified by profession or role in the results section in order to give each one an equal voice and status towards the reader.

Two FGDs were conducted with mothers at the inpatient clinic and two FGDs with mothers at the outpatient clinic. 48 mothers were recruited on site for a FGD after a regular teaching session that is part of the nutrition program. The health worker in charge on the day of the FGD identified mothers who had young infants (under 6 months) being treated for acute malnutrition or whose child had started treatment in that age group (purposive sampling), and asked them individually to participate (Fig. 1). The researchers aimed for a group size of 8 to 12 mothers. This group size was chosen because a larger group would have made discussion less dynamic and a smaller size may have limited the number of perspectives. Purpose and general information about the study were explained to the entire group. Those who agreed to take part signed a consent form and were directed by the research assistant to the court yard of the clinic, to start the FGD. Demographic data of the participants was not obtained. This added a level of confidentiality particularly because of the small sample size.

The FGDs were conducted in French, with simultaneous translation into Wolof by the research assistant. Each FGD took about 30 minutes to complete. Questions were similar to the ones in IDIs but put in a story. For example: “Your cousin has a baby that is not growing well, he is very small. What would you recommend her to do? Where to go? Why?”. Mothers were also invited to tell their own stories on the topic until no new aspects were mentioned (saturation).

### Data analysis

IDI and FGD transcriptions were analysed by giving codes to quotes. To code a deductive coding was first performed manually, using only the 3 key factors: access, quality of care and community engagement. Then, each quote was given a sub-code, that emerged from the transcripts. For coding Microsoft Word was used. Coded quotes were copied into an excel sheet, organising them in groups of similar codes. This was an iterative process and some codes could be grouped and merged. For example quality of care- illness and quality of care- protocols merged into quality of care- medical care. Quoted phrases were translated into English.

## Results

**Fig. 2:**
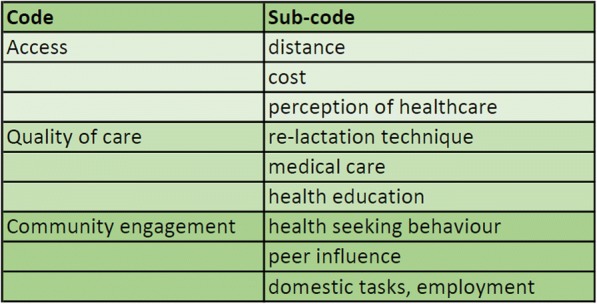
Codes and sub-codes

The health workers who participated included three nurse assistants, three nurses, two midwives and four doctors. The participants in the FGDs were mothers of infants that were in the treatment process at that moment. As mentioned above, demographics of the participants were not investigated, but from the discussions we noted two relevant facts. Most mothers were not employed outside the home and a number of them had no formal education. Within the 3 key factors, 9 facilitators and barriers have emerged from the data (Fig. 2). Distance, cost and perception of healthcare (Access), re-lactation technique, medical care, health education (Quality of care), health seeking behaviour, peer influence and domestic tasks (community engagement).

### Key factor 1 Access

#### Distance

Almost all participants, both mothers (in all 4 FGD) and 11 of the 12 health workers, mentioned distance from home to a health care facility as an important access barrier to care for malnourished infants. Many mothers in the inpatient group had travelled a large distance to get to the health service. The rooming-in option at St. Martin was preferable for those who did not have family in the capital. An outpatient treatment approach, involving weekly visits, was mentioned as convenient for women who came from the neighbourhood in which the clinic was located. Three health workers from Keru Yakaar mentioned that a large distance made it much harder to provide follow up for their patients.

*“Distance plays a role because the majority of the mothers who come here are not from the neighborhood, they come from far away and that’s a problem.” (a health worker, IDI_7)*

*“If there would be a service like this in my neighborhood, I would not have to do all this traveling to come here, I would be close to home.” (a mother, FGD_4)*

#### Cost

The cost of treatment was a recurrent barrier in both treatment approaches. Women make their financial calculations as soon as a treatment is offered. Health workers noticed that even poor women sometimes manage to obtain the means to cover all costs if they highly value the treatment of their sick child. Inpatient care is perceived by mothers as more expensive, which is a barrier in seeking care. Most women who seek care expect to receive milk for their infant. Many mothers had already given a milk supplement before visiting a health centre. The availability of a milk supplement was an attractive factor for a health centre. Health workers were worried that inappropriate or too much diluted milk had actually contributed to the infants’ malnutrition.

*“She (the mother) can tell she wants to (start treatment) but it is the numbers that come immediately into her head that make her decide not to when she finds it expensive.” (a health worker, IDI_8)*

*“It cannot be a matter of lack of money, because you will surely be able to manage and even if you have to borrow money to care for your child you will do so.” (a mother, FGD_3)*

*“Often when I ask them questions, I note that they have already bought milk somewhere. Sometimes it is milk from the pharmacy but often it's cheap milk from a shop.” (a health worker, IDI_5)*

#### Perception of healthcare

The way that health care is perceived by the mother was a recurrent and often an access barrier. Health workers explained that when receiving patients they had an influence on the woman’s decision as to whether she would stay or not. Mothers explained that if a health worker could convince her a certain treatment was really going to help her infant, other access barriers could be overcome more easily. In general, hospital care is perceived by mothers as only for serious cases, forming a barrier for some of them. Health workers mentioned that if mothers were familiar with outpatient primary care services because of prenatal care and vaccinations offered there, trust could be generated more easily. Some women though, expressed not having much confidence in health posts nearer to them.

*“Sometimes in the social reality the hospital or health facility is not well seen, that means that if a child is hospitalized for a mother it is synonymous to having a serious disease, that can even cause death, so in general people try to avoid hospital care.” (a health worker, IDI_6)*

*“But when the health workers say the child will be hospitalized they (the mothers) will tell you they have no one to leave at home but after explaining what the health worker is going to do they can accept and sometimes the father too.” (a health worker, IDI_11)*

*“It’s not any health post that one trusts because when you go to some health post your child will not be cured. This was my case.” (a mother, FGD_2)*

### Key factor 2: Quality of care

#### Re-lactation technique

Refeeding the infant was the main topic discussed during IDIs and FGDs concerning the quality of care. The supplementary suckling (SS) technique, meaning giving milk supplement by attaching a tube to the breast of the mother, had been abandoned in St Martin mainly because of practical difficulties experienced by the staff. Cup and spoon feeding was the re-lactation technique promoted in both clinics. The importance of the mother-child attachment during re-lactation treatment was mentioned by 5 out of 12 health workers and was elaborated in 3 FGDs. Health workers stated that home-based care can stimulate mother-child affection in the sense that mothers stay in their natural environment. Mothers mentioned the importance of maternal warmth, even when the infant receives a milk supplement. Most hospitals in Senegal do not offer rooming-in, but St Martin does. Health workers at St Martin thought that in inpatient care re-lactation would be more effective because of closer supervision with both breastfeeding and supplementary feeding. They mentioned the risk that errors or continued bottle feeding are not corrected in a timely manner when the mother has outpatient appointments. All 6 health workers at Keru Yakaar and mothers in 2 FGDs testified cup and spoon feeding to be a useful method in an outpatient setting .

*“From my point of view, it's better to keep them because in general the hospitalized child will better follow the treatment protocol and therefore he recovers faster than children who come to outpatient.” (a health worker, IDI_7)*

*“What causes problems is when the person returns home and does not give the treatment correctly, in that case the treatment duration will be longer.” (a health worker, IDI_2)*

*A mother: “I prefer keeping my baby home, because at home I will take good care of my child by giving him milk with a spoon and cup and also by breastfeeding. I can stay close to my child and he will feel my maternal warmth.”* (a mother, FGD_1)

#### Medical care

While mothers had much to say about access factors, it was mainly health workers who commented on medical care for infants. Complications such as pneumonia or severe oedema were reasons why the health workers recommended hospital treatment. Three of the health workers specifically mentioned congenital illnesses as underlying causes of malnutrition that need specialist care. From health workers’ perspective, most medical conditions in malnourished infants are minor and can be treated following outpatient treatment protocols. Another argument mentioned was that there are simply not enough hospitals to provide inpatient care. Mothers had the idea that their baby would recover quicker when medicines were administered under supervision of a health worker. Some health workers (4 out of 12) thought that compliance with treatment would be worse at home.

*“I prefer to hospitalize my child because in the hospital, the child will be well controlled and also followed and will have good care and will be restored and I go home.” (a mother, FGD_1)*

*“I do not think there is any difference (in medical treatment), because what we give to the outpatients we also give in hospital, so I think it can be done either way.” (a health worker, IDI_1)*

*“It is true that there are not enough hospitals and even in hospitals there are not enough paediatricians, we cannot decide to hospitalise every case with malnutrition even without the complication. You have to look case by case, so that only the complications stay in hospital.” (a health worker, IDI_6)*

#### Health education

Educating mothers on nutritional practices related to health was viewed by all interviewees as an important aspect of health care. Health education was said to be more intensive during inpatient care, because health workers had more individualized time with the mothers. One St Martin health worker admitted that in daily practice the health workers do not always have sufficient time to provide this supervision. During outpatient care, health education needs to take place during the regular visits and some health workers mentioned a time constraint. The lack of home visits was mentioned by them as a barrier to good health education. Breastfeeding education was mentioned as the main education topic. The importance of breastmilk for the infants was said by 11 out of 12 health workers to be underestimated in communities. Mothers easily start bottle feeding or give porridge in an early stage and misconceptions about young infant feeding are frequent. The majority of health workers stated that breastfeeding education was lacking and exclusive breastfeeding was hardly ever met as a discharge criteria.

*“Here they help us, we are also taught how to take care of our child even when coming home afterwards.” (a mother, FGD_3)*

*“For certain people the explanation needs to take place inpatient because a soon as they come home after having heard a teaching they forget quickly. Sometimes when mothers come back the next day and when you ask questions they will tell you that they have all forgotten.” (a health worker, IDI_11)*

*“Many times, breastfeeding is a problem, mothers just need to give the breast, but often moms do not even know how to breastfeed their children.” (a health worker, IDI_9)*

### Key factor 3: Community engagement

#### Health seeking behaviour

In all four FGDs mothers testified they had been coming to the clinic because of a neighbour’s recommendation. Mothers, whose child had been well treated for malnutrition, shared their experiences with their community. The family, in particular the father of the baby and the mother-in-law, were said to be the ones identifying underweight. Some women came with stories of babies from undesired pregnancies where a family member brought the child to the clinic instead of the mother because of shame. In other cases, women said they needed permission from their husband or other household members in order for their infant to be admitted. Although mothers themselves did not mention this, a few health workers disclosed that religion and traditional medicine are of great importance in the early life of a new-born. They said health care is often first sought from religious leaders instead of at the health service.

*“Normally when the child takes the breast it must gain weight but when there is no growth another mother in the house can know that there is a problem because the child needs to grow normally.” (a mother, FGD_2)*

*“Because I knew of a child who had been here for treatment. That’s why when I found out that my child was malnourished I came straight here.” (a mother, FGD_2)*

*“When I was offered to stay I went to ask my husband. He refused because there is work at home and I live with his co-wife, so my husband preferred me to do outpatient treatment because of the work at home.” (a mother, FGD_1)*

#### Peer influence

Community support differs greatly from place to place. Health workers said they took this into account when deciding for inpatient or outpatient treatment. When the community is not supportive, this will be a barrier and it might be better to admit an infant with its mother until rehabilitated. Some health workers said inpatient treatment can protect the mother from the influence of misconceptions and malpractices around breastfeeding. Mothers admitted peer pressure to give water or food supplements to malnourished infants can be high. But inpatient care carries the risk of relapse when community habits do not change. Some health workers explained that the community has the potential to be very much involved in home-based treatment. It can support mothers during treatment of the new-born and with breastfeeding. Only a few health workers said this was currently happening though and several of them from both clinics underlined the lack active community or peer involvement.

*“When he was born, my child had a good weight and he took the breast well, but I lost weight so my husband told me to stop giving the breast to the baby so I took the bottle.” (a mother, FGD_3)*

*“I was asked (by my neighbors) what I give to the child as food and I say nothing and they recommended the milk and then I first bought the milk and give it to the child.” (a mother, FGD_3)*

*“This (community involvement) is not the same for everyone. There are families that help and are encouraging, they are with you wherever you go. Other families are different and if the child is ill they do nothing.” (a health worker, IDI_2)*

#### Domestic tasks, employment

During FGDs, domestic tasks were often mentioned as a barrier for inpatient care. Mothers in all 4 FGDs mentioned the fact that domestic tasks can be continued is a facilitator for outpatient care. Several working mothers also preferred outpatient treatment so they could come before or after work to the clinic. Most mothers preferred to be home especially for breastfeeding the child. Health workers stated that mothers who did manage to stay for inpatient care often saw it as an advantage to be able to fully focus on their child without distraction

*“I chose the outpatient care because I have to do work at home and after that I can come.” (a mother, FGD_4)*

*“Sometimes there are moms who have work at home and forget their child, in this case it is the moms who does everything in the house and also some of them having trouble giving the breasts to their child regularly and they forget.” (A mother, FGD_2)*

*“When the mothers are here, they have much more time to care for their child and they respect the feeding hours. They are being monitored every three hours the child they must nourish their child.” (a health worker, IDI_10)*

## Discussion

**Fig. 3:**
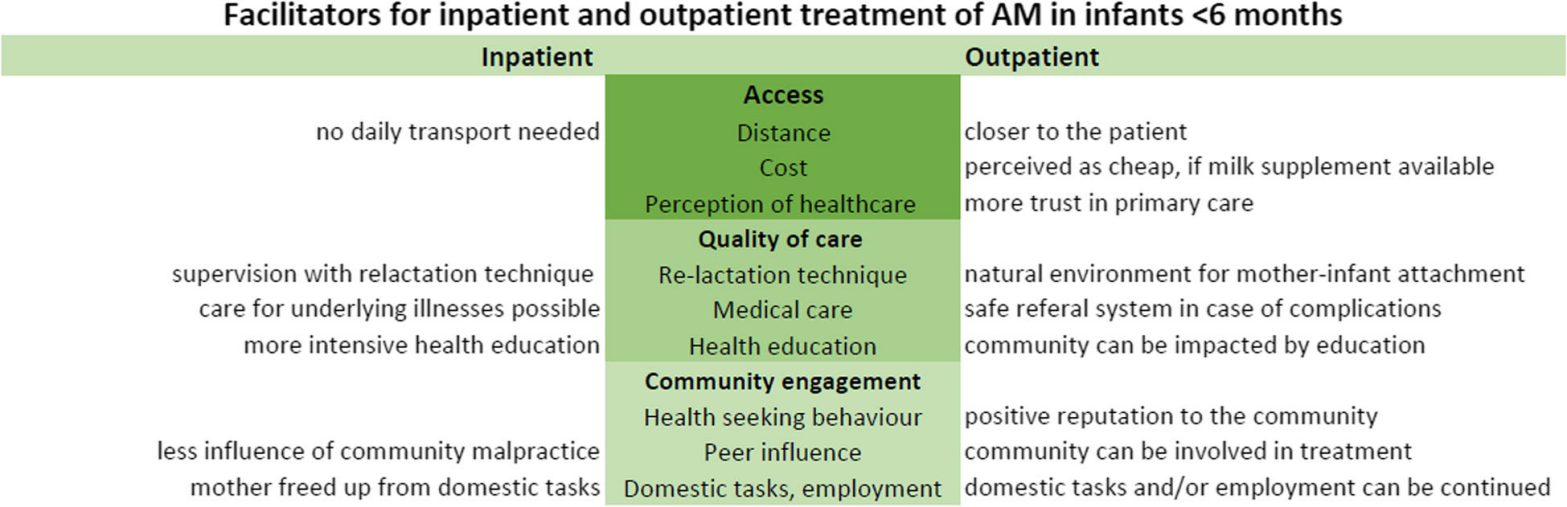
Facilitators for inpatient and outpatient care for infants <6 months with AM

There is limited evidence on facilitators and barriers to treatment approaches for malnutrition in infants <6 months. WHO recommends an inpatient approach [5], but in practice both inpatient and outpatient care are being applied, as seen in the two clinics where this study was conducted. Treatment choices vary, depending on the perspectives of health care workers and mothers of malnourished infants. This qualitative study, using both IDIs and FGDs, revealed some of those perspectives. The key factors access, quality of care, and community engagement framed 9 facilitators and barriers presented by health workers and mothers. In Fig. 3 some of the main facilitators for each treatment approach are listed.

### Key factor 1: Access: distance

Distance was frequently mentioned as an access barrier. This is understandable given that health services offering care for malnourished young infants are scarce in Senegal. Because treatment is generally intensive and long, there is a risk for loss to follow-up when health services are too far from home. A rooming-in service is one solution, but if women can choose they would rather have an outpatient service close to their home.

### Key factor 1: Access: cost

Cost for care of a malnourished infant is another barrier to care in Senegal. Most mothers seek care at a clinic because they think their child needs a milk supplement and infant formulas sold at the shops become too expensive. The lack of an affordable milk supplement at primary care level forms a current access barrier. In Senegal, the milk supplement F-100 is provided for free by the government but exclusively in inpatient services. Nevertheless, care in hospitals is generally perceived by patients to be more expensive. Keru Yakaar is one of the few clinics that offer infant formula for a low price.

### Key factor 1: Access: perception of healthcare

The lack of trust in a health service appeared to be of great importance and other barriers seemed easier to overcome. Building a reputation of trust often starts at entry level. Guerrero found that lack of good reception or earlier experience of rejection at a health service was one of the main barriers to access to outpatient nutrition programs [23]. Inpatient care was perceived by mothers in this study to be only for children who were seriously ill. When inpatient care is the only available option, mothers wait until the situation is serious before seeking care [24]. Outpatient services as first point of care, with possible referral, could remove this barrier. When \provided at a primary care level, a nutrition program could be embedded in the existing health care, as are prenatal care and vaccinations, thus generating trust.

### Key factor 2: Quality of care: re-lactation technique

An effective re-lactation technique is essential for giving good quality care. The SS method is the recommended inpatient re-lactation method for malnourished infants in the WHO protocol [5]. Field reports show good results, but say that this method requires intensive guidance of both mothers and medical staff [25, 26]. The cup and spoon-feeding method has the advantage that it can be used in the outpatient setting. This current study shows good feedback on this method. It is mentioned as an option in the new WHO guideline on breastfeeding support in health facilities [27]. In refeeding the infants, mother-child attachment is an important aspect, which is often underestimated in nutritional care [8]. The MAMI project concluded that children and young infants receiving stimulation during treatment for severe malnutrition have significantly superior intellectual development than the control group [8]. Rooming in is a WHO recommended solution [27]. Home-based treatment would be naturally beneficial regarding mother-child attachment.

### Key factor 2: Quality of care: medical care

Good quality medical care in an outpatient nutrition program for young infants is thought to be ensured, if treatment protocols are followed and an appropriate referral system is in place. Following the WHO guideline [5], acute malnutrition among infants <6 months can be treated in an outpatient setting, but infants with complications or underlying illnesses need to be referred to hospitals. A few health workers in this study suggested that nutritional treatment could happen in an outpatient program, while simultaneously treating some underlying illnesses during paediatric appointments at a hospital.

### Key factor 2: Quality of care: health education

Educating mothers on nutrition and health is a facilitator for good quality care, but often insufficient due to time constraints and a lack of home visits. Especially breastfeeding counselling appeared to be a huge need that could often not be met in a clinical setting. This weakness is reflected in the health workers’ concern about the infants still needing milk supplement at discharge. Breastfeeding counselling can be done in outpatient settings [28]. Aidam et al. studied a healthy population and found a 100% increase in the rate of exclusive breastfeeding by a combination of educational sessions during regular prenatal visits and home visits [29]. This could be applied and evaluated in the Senegalese context.

### Key factor 3: Community engagement: health seeking behaviour

In Senegal, family members like the grandmother are involved in recognizing the problem of malnutrition, and influence the type of care to be sought. Religious leaders have a strong influence on health seeking behaviour, especially concerning new-borns. Fathers or other male household members are the decision makers for the financial aspect of care. Because outpatient care is physically closer to those key community figures, seeking care will be facilitated by communication and interaction with them. Guerrero describes success of a community based nutrition program as partly defined by how well it is known in the community [23]. Good treatment outcomes will result in a clinic’s good reputation, which could lead others to seek help earlier.

### Key factor 3: Community engagement: peer influence

The negative influence of peers was mentioned more frequently in this study than the possible benefits. Misconceptions about breastfeeding, for example, were said to influence a mother and child in treatment; inpatient care would allow the mother to be more detached from the community to learn new feeding practices. Ashworth underscores this by showing that hospital based care showed less risk of relapse [30]. On the other hand, outpatient care has the potential of favouring community involvement when mothers become initiators of change in their community by sharing new feeding practices with neighbours and peers. A study in Bangladesh showed the importance of peer support in breastfeeding counselling. A series of counselling visits to healthy mothers with their new-born babies dramatically improved exclusive breastfeeding rates [31]. Active involvement of the community is currently lacking in both clinics, which is a weakness.

### Key factor 3: Community engagement: domestic tasks, employment

Domestic tasks form a main barrier for either treatment model. Mothers frequently refused treatment or defaulted because of domestic tasks. Often it was not the mother making this decision, but the husband or the community as a whole. Outpatient care was most preferred by mothers allowing them to continue their domestic, or paid work while going for regular visits. Health workers were concerned that mothers would easily be distracted by domestic work when treating them at home. Inpatient treatment allows the mother to fully focus on the treatment without interruptions.

### Limitations

This study has several limitations. Only mothers were interviewed, while male care givers perspectives could further enrich the findings. Another limitation was the fact that the translator during FGD was a nurse at the nutrition department She knew some of the participating mothers in this clinic, which could have influenced participants’ responses, not wanting to hurt the feelings of their treating nurse. On the other hand, our experience with patients had shown us that questions from strangers raised suspicions. As a health worker herself, the translator was able to create an atmosphere of trust and free exchange. The women were invited to speak out of their experiences, tell their stories, and not criticise the health care as such. Her role was seen more as facilitating rather than a limiting because of the nature of the discussion. Applicability is an issue, this is a study conducted in urban Senegal, while health workers and mothers opinions might be different in rural setting or in neighbouring countries. The 9 factors are very general though and can probably still be transformed into recommendations in a different setting.

## Conclusions

Within the global health issue of maternal and child malnutrition, AM of infants <6 months has often received less attention, even though the prevalence in this age group is comparable to or higher than in older children. This study gives insight into mothers’ and health workers’ perspectives regarding inpatient or outpatient treatment for acutely malnourished young infants using qualitative data from two clinics in urban Senegal. Within the 3 key issues of a successful community based nutrition program (Access, Quality of care and Community engagement), 9 facilitators and barriers were identified that play a role in choosing an appropriate treatment approach. Although inpatient care is the actual standard, this study shows that outpatient, community based treatment demands serious consideration. Outpatient care could facilitate access, given an affordable milk supplement is available. Quality of care can be guaranteed using an appropriate re-lactation technique and a referral system for complications. The community has the potential to be much involved, but more attention is needed for breastfeeding education. Further research is needed to gain evidence on specific treatment aspects, efficacy and cost effectiveness.

## Abbreviations

AM: Acute Malnutrition
ENN: Emergency Nutrition Network
FGD: Focus Group Discussion
IDI: Individual Depth Interview
MAMI: The Management of Acute Malnutrition in Infants under six months
MUAC: Mid Upper Arm Circumference
RUTF: Ready to Use Therapeutic Food
SAM: Severe Acute Malnutrition
SS: Supplementary Suckling
UN: United Nations
WHO: World Health Organisation
